# Developing forensic patient-oriented research guidelines: a rapid review using an integrated knowledge translation approach

**DOI:** 10.3389/fpsyt.2026.1805912

**Published:** 2026-07-02

**Authors:** Christopher Canning, Cara Evans, Sevil Deljavan, Kristy Allen, Laura Justine Chouinard, Amber Scott, Elnaz Moghimi, Konstantina Poursanidou, Stephanie Junes, Jolene Wintermute, Kayla Zimmermann

**Affiliations:** 1 Waypoint Research Institute, Waypoint Centre for Mental Health Care, Penetanguishene, ON, Canada; 2 Department of Psychiatry, University of Toronto, Toronto, ON, Canada; 3 Patient/Client and Family Council, Midland, ON, Canada; 4 Department of Psychiatry, Faculty of Health Sciences, Queen’s University, Kingston, ON, Canada; 5 Independent Researcher, Preston, United Kingdom

**Keywords:** forensic mental health, guidelines, integrated knowledge translation, patient-oriented research, rapid review

## Abstract

This paper reports findings from a rapid literature review that informed new guidelines for conducting patient-oriented research in forensic mental health settings. The project adopted an integrated knowledge translation approach at a mental health hospital in Ontario, Canada, engaging a project team that included current forensic patients, hospital staff, and members of an international community of practice. Sources were identified through nine academic databases and targeted grey literature searches, screened independently by two reviewers and extracted using a structured template guided by an *a priori* framework developed with patients and staff at a knowledge exchange event. Findings were iteratively refined through a patient advisory group, an implementation study, ethnographic observations, and related integrated knowledge translation activities conducted alongside the review. Together, 31 academic and grey literature sources informed a framework organized around five core dimensions: 1) Resourcing, orientation, and training; 2) Confidentiality, consent, and compensation; 3) Relationships, shared understanding, and support; 4) Levels of engagement; and 5) Evaluation and sustainability. Guided by cross-cutting principles common among participatory mental health research, such as dignity, trust, respect, and a commitment to redressing power and attending to forms of epistemic injustice, the guidelines respond to distinctive constraints of forensic environments while highlighting opportunities to promote authentic co-production and sustain patient involvement in research. Recommendations include dedicated resources and capacity-building for patients; relational, ongoing consent practices co-developed with patients; flexible patient researcher roles with fair, paid compensation; and sustained institutional support for participatory practices. We call on forensic hospitals and secure settings to adapt and evaluate these guidelines and to invest in expanding patient leadership to advance the field.

## 1 Introduction

Patients in forensic mental health settings are rarely actively involved in research that affects them, but exciting examples of patient-oriented research (POR) in these settings continue to emerge. A synthesis of key practices and principles is needed to support learning across this emerging field and to enable further iteration and growth. This paper draws on a rapid literature review conducted to inform guidelines for POR in forensic mental health settings. This review was conducted as part of a broader project to establish participatory research approaches and a program of patient-oriented forensic research in a specialized hospital setting.

The forensic mental health care system serves people at the intersection of the criminal justice and mental health systems, including those found Not Criminally Responsible (NCR) or unfit to stand trial (UST) due to mental illness. Patients in forensic mental health care settings frequently experience layered stigma and intersecting forms of marginalization, including early childhood trauma (1). Forensic mental health care services demand a balance of rehabilitation, recovery, and risk management that creates unique ethical and operational challenges (2). Institutional rules and security practices can entrench power imbalances, restrict autonomy, and complicate efforts to build trust and deliver recovery-oriented care. When people are detained or involuntarily treated, authentic power-sharing, non-coercion, and collaborative decision-making become particularly difficult in everyday practice. Positively, a shift is underway in forensic mental health settings toward co-designed and recovery-oriented models and the active involvement of patients in decisions about their care. Recovery-oriented models such as CHIME-S reflect this shift by adapting the CHIME framework to the safety and security demands of forensic settings (3).

Patient-oriented research seeks to engage patients, family carers, service providers, and other system interest-holders (4) as partners and leaders in the research process, with the aim of improving health systems, care experiences, and ultimately health outcomes (5). POR is informed by traditions of participatory action research (PAR) and community-based participatory research (CBPR) but focuses specifically on health care settings (6). Survivor research and Mad Studies traditions offer critical perspectives on these participatory approaches, foregrounding the epistemic authority of people with lived experience of mental illness and challenging forms of institutional participation that risk reproducing power asymmetries (7–11). Faulkner (7), a survivor researcher, is among few researchers to connect survivor research to forensic mental health contexts, and her work highlights the ethical stakes of participatory research approaches with patients living with mental health-related disabilities in forensic settings. Like PAR, CBPR, and these more critical epistemologies and methodologies, POR emphasizes meaningful, sustained involvement across all stages of research, from priority setting and conceptualization to knowledge translation and implementation (6). Ideally, it draws on the principles of co-production, treats lived experiences of marginalization as essential to creating experiential knowledge that can meaningfully effect change (12) and deliberately addresses power dynamics to support equitable decision-making in research and practice. Despite these ideals, POR has been criticized for failing to uphold them in meaningful ways. Criticisms include neglecting the deeper power relations and structures inherent in biomedical and health research practices, reinforcing tokenistic patient involvement, and co-opting emancipatory practices to maintain the status quo (13, 14).

It is important to note that in different contexts, such as the United Kingdom and several European countries, POR is referred to as service user involvement in research, Patient and Public Involvement (PPI), or co-produced research. In a Researcher’s Guide to Patient and Public Involvement, the authors argue that:

Patients and members of the public bring an ‘expert’ insight into individual research projects because of their experiences of living with a particular condition or using health services. Involving Patient and Public Involvement contributors in research allows ‘the color and nuance and diversity’ of different types of knowledge to be valued and to improve research. Involving those with ‘lived experience’ enables researchers to access a fuller understanding of the conditions being studied and may help generate research which is more meaningful (15).

Similarly, a UK report examining the values of co-production identifies a range of values and principles that ideally underpin co-produced research. These include, among many: working towards social justice, sharing power, embracing diversity in perspectives and experiences, valuing learning and personal development, challenging both the status quo and the perspectives of all co-producers through continuous reflexivity and questioning, embracing complexity to support system change, and being attentive to the contexts in which research is being co-produced (16).

A limited but growing body of work suggests that these aspirational principles and participatory research approaches in forensic mental health settings may improve patient safety and dignity and promote recovery trajectories (17–21). Partnering with forensic patients and family carers through peer research teams or patient advisory councils, for example, may help to address structural marginalization (22). Collaborative decision-making among patients, clinical staff, security staff, and researchers can also democratize research practices and knowledge production in forensic settings (23).

Despite the extensive use of participatory research approaches in other mental health settings (24), guidance on implementing and conducting patient-oriented and other participatory research methods in forensic mental health settings remains scarce, as does evidence on their short- and long-term challenges or benefits. Evidence-informed participatory research frameworks tailored to forensic mental health settings have not been established. This might be due to the dominance of risk-focused approaches over recovery-oriented ones, and the persistent discrimination toward individuals with mental illness diagnoses involved in the criminal justice system. Additional challenges include risks of tokenism and difficulties establishing and sustaining trust between forensic mental health patients and researchers, as well as pronounced power imbalances between patients, on the one hand, and researchers and clinical staff, on the other (18, 20, 21). Security protocols, risk management priorities, and restrictive practices further shape what patients feel able to share with researchers and how their input is interpreted by healthcare providers and researchers (25, 26). Upholding core principles of genuine collaboration and power sharing is, therefore, particularly challenging when patients are detained or receiving involuntary treatment within such environments.

To address the aforementioned evidence gap, and to support what we hope to be more authentic participatory research practices in forensic mental health settings, the project discussed in this paper aimed to develop comprehensive, context-specific guidelines for conducting POR grounded in existing literature and the lived experiences and expertise of patients, staff, and researchers in forensic mental health settings. The project team adopted a multi-component, integrated knowledge translation (IKT) approach, explained further below. This paper presents findings from a rapid literature review on participatory research in forensic mental health. We selected a rapid review because it is well-suited for contexts in which timely synthesis is needed, the research question has been clearly defined, and the overall scope is sufficiently focused to allow streamlined yet rigorous methods (27) – in our case, for informing guideline development. This review was based on prior project activities and integrated with prior research findings to develop guidelines.

The guidelines described in this paper are publicly available here (28). We have also published what we called a “how to” handbook to accompany the guidelines. The handbook is also available online here (29). It is based on the guidelines and offers practical advice for anyone who wants to design, carry out, or participate in POR in forensic mental health environments. While this review paper reports on the guidelines document and its foundational research, the handbook offers more focused, step-by-step tools to support research teams.

## 2 Materials and methods

### 2.1 Project team and setting

The project took place at Waypoint Centre for Mental Health Care (Waypoint) in Ontario, Canada. Waypoint offers the province’s only high-secure program with extensive security measures and high staffing ratios that serves patients who require intensive mental health treatments and other psychosocial supports. The multidisciplinary project team included researchers, a knowledge translation and implementation coordinator, a medical librarian, representatives from the Patient/Client & Family Council (PCFC), current forensic patients, and members of an international community of practice developed by the research team at Waypoint. This team composition brought together theoretical, methodological, clinical, implementation, and lived experience perspectives on mental illness and marginalization to guide the project.

### 2.2 Overall approach

The literature review presented in this paper was one component of a broader project, which adopted an integrated knowledge translation (IKT) approach. In IKT, end users and researchers collaborate throughout the research process, from defining the research problem and selecting a methodology to collecting and analyzing data and disseminating findings (30). IKT is characterized by a focus on co-creating applied knowledge and by including health system decision-makers as collaborators (30, 31). Patients are also end users of knowledge who may be positioned to both contribute expertise and act on research findings to effect change (32). While IKT departs from other participatory approaches in its focus on system impact rather than justice or emancipation (30, 33), it shares with them a commitment to genuine engagement, multi-directional learning, reciprocity, trust, and actionable knowledge (33). Epistemologically, IKT has been described as “neutral” and amenable to integration with multiple methodologies and philosophical stances (33). Adopting an IKT approach within our project team enabled us to engage with multiple knowledge end users and refine our questions, objectives, and approach to meet emerging project needs.

The wider project encompassed multiple elements and strategies that integrated local knowledge and lived experience/expertise of mental illness and marginalization, national and international experiences, and academic literature. These elements and strategies included:

- Receiving mentorship from POR experts at the University of Saskatchewan, who provided feedback on our overall approach and development of the guidelines.
- Leading extensive engagement sessions with patients and staff in a forensic setting to develop trusting relationships (34). These involved researchers visiting patient programs and attending community meetings over the course of a year before any formal research or guideline development started. During that year, researchers made unstructured visits focused on getting to know patients and building trust. We documented field notes and reviewed them during team meetings. The notes helped to generate insights that informed the guidelines and our reflections on positionality, explained further below.
- Conducting an implementation study that used qualitative interviews examining Waypoint staff and patient perspectives on barriers and facilitators to POR in forensic settings, guided by the Consolidated Framework for Implementation Research (CFIR) (26).
- Hosting a knowledge sharing event to share learning emerging from the above processes, to identify core guideline dimensions, and to clarify next steps for practicing/conducting forensic POR at Waypoint (35).
- Reflecting on ethnographic observations of the project team’s navigation of power dynamics in the early stages of the project (36).
- Convening a research advisory group of forensic patients and putting together an implementation team, consisting of a forensic security officer, a spiritual care staff member, allied health professionals, and clinical staff champions. The research advisory group included three forensic inpatients who met with the project PI and research analyst every two to three weeks for six months through guideline development. Seven forensic patients were engaged in a separate validation process to review and provide feedback on the draft guidelines, which took an additional six months.
- Engaging with an international community of practice to gather examples of emerging best practices and shared challenges from partners from the UK, Germany, Belgium, Switzerland, and the USA.

The rapid literature review presented here was informed by earlier activities and initiatives in the wider project. For instance, a framework that was developed at the knowledge exchange event provided an initial structure for categorizing findings from the literature review. The research advisory group of patients, convened within the larger project, provided feedback to support the translation of the review findings into guidelines. Findings from earlier project stages, such as the implementation study described above, were also integrated with structured review findings. This iterative approach enabled us to integrate end-user knowledge, contextual considerations, and academic literature to develop a set of POR guidelines tailored to forensic mental health care environments. As a rapid review, this paper departs from a systematic review methodology in several ways. We did not use a PICO (Population, Intervention, Comparator, Outcome) framework, as the guiding research question and *a priori* roadmap framework provided the structure for data extraction and analysis, and the review did not aim to assess intervention effectiveness. We did not register a review protocol, consistent with rapid review practice. These methodological choices align with rapid review approaches and reflect the pragmatic nature of this project (27).

### 2.3 Ethical considerations and reflexivity of the research team

The broader project received approval from Waypoint’s Research Ethics Board (HPRA 23.08.21). All patient-facing materials were drafted in plain language and refined with feedback from patient advisors to promote understanding and meaningful engagement. Plain language materials explaining POR were also distributed and informally tested during community visits before being used among the patient advisory group. This allowed us to make sure that the concepts and language we used were accessible to patients across a range of literacy levels.

The project team’s diverse expertise in forensic mental health, community engagement, participatory research, and knowledge translation brought important strengths to the project but also raised questions concerning potential gaps. To address these gaps, the team held regular reflexive discussions about how institutional roles and disciplinary backgrounds might shape our approach and the interpretation of findings. Emerging frameworks and draft guideline components were presented to patient advisors and frontline clinical staff with explicit invitations to critique content, highlight jargon or unclear concepts, and identify missing priorities. These ongoing dialogues helped the team remain attentive to issues of positionality and contributed to guidelines that reflect the needs and insights of people with lived experience of mental illness and marginalization in forensic settings.

We also acknowledge the gap between the participatory ideals articulated in the guidelines and the level of involvement we were able to achieve. Patient involvement in this project was, we admit, primarily consultative. Patients reviewed draft content, identified inaccessible language, and validated emerging recommendations or helped refine them. This is genuine and valuable engagement but does not fully correspond to the co-production or patient-led research we draw from and to which we aspire. We are aware of this, and the guidelines caution against reproducing tokenism and co-optation that have been identified repeatedly as a risk in participatory research with marginalized populations (7, 9, 11, 14).

That said, we monitored co-optation and tokenism closely. The patient advisory group’s involvement was structured to give patients meaningful influence over the content and direction of the guidelines. For example, patients raised concerns related to sharing information in their roles as research team members that could impact clinical decisions or Review Board hearings, an issue that was unrepresented in the literature that became a recommendation in the guidelines. We also co-developed a shared goals agreement and formalized principles for working together on research teams, which became recommended tools in the guidelines. One patient partner was involved throughout the full project and co-authored a related publication with the research team on building trusting relationships in restrictive environments (34). All patients were paid $30/hour for their time on the project.

Using the Saskatchewan Centre for Patient-Oriented Research (SCPOR) level of engagement tool, patient involvement in this project reflects levels two and three (consultation and involvement) (35). We did not reach the collaboration or empower levels that characterize power sharing and co-production, and we recognize that in secure forensic settings, full empowerment may not be structurally achievable at this stage. Nevertheless, we framed our approach as a starting place and foundation. The expansion of patient leadership and power sharing roles remains a central goal of future work, and we recognize the importance of naming, agreeing on, and openly discussing levels of involvement and expectations between researchers and patients on an ongoing basis.

### 2.4 Study eligibility

The review of the literature was guided by the following research question: *What tools and resources exist for conducting patient-oriented or participatory research in forensic settings, and how have they been applied*?

The review included academic and grey literature that offered guidance, tools, or resources for conducting patient-oriented or participatory research and for engaging patients in secure, inpatient forensic mental health settings as research partners or collaborators. The review excluded studies focused on outpatient or community forensic contexts, family carers, justice-involved individuals without forensic mental health contact, and youth under 18. Furthermore, it excluded qualitative and quantitative studies without patient or service user partnership or guidance on patient involvement. Inclusion and exclusion criteria are summarized in **Table 1**.

**Table 1:** Inclusion and exclusion criteria.

Inclusion	Exclusion
**Participants and context** • Literature that involves the engagement of inpatient forensic service users as partners in the research process. • Conducted in inpatient, secure forensic mental health care settings. **Types of research** • Patient-oriented research, participatory research, or other patient engagement strategies in research. • Use of POR, participatory approaches, or related strategies may be either explicit (i.e., the authors state this is what they are doing) or implicit (i.e., authors do not use these phrases but nonetheless describe partnering with patients through any or all stages of research). • Engagement may take any form along a continuum spanning from limited involvement to patient leadership. **Type of sources** • All, including research and quality improvement reported in original research studies, all forms of evidence synthesis, discussion papers, conceptual and theoretical papers, book chapters, dissertations, theses, and grey literature, including toolkits, guidelines, policy papers, and related sources.	**Participants and context** • Literature that exclusively focuses on patients in forensic outpatient or community-based settings. • Literature about family carers as partners in the research process. • Literature about justice-involved individuals who have not had contact with the forensic mental health care system. • Studies that involve participants under the age of 20 involved in the youth criminal justice system. **Types of research** • Research that is not patient-oriented or participatory but rather collects data from or about forensic patients through surveys, interviews, focus groups, or other qualitative or quantitative methods. • Literature not explicitly making recommendations or providing guidance about how a team could actively involve patients in forensic research. **Type of sources** • Posters, abstracts, or research protocols about patient involvement in forensic research.

### 2.5 Search strategy and data sources

Our search strategy aimed to identify both published and unpublished primary studies, reviews, conceptual and theoretical papers, book chapters, theses and dissertations, and grey literature. We searched Medline (Ovid), PsycInfo (Ovid), Embase (Ovid), CINAHL (EBSCO), ASSIA (ProQuest), Criminal Justice Abstracts (EBSCO), Sociological Abstracts (ProQuest), ProQuest Dissertations & Theses, and Google Scholar, with no restrictions on publication date or language (see **Appendix A** for a sample search strategy). An initial search in Medline (Ovid) identified five studies that met the inclusion criteria and keywords and index terms from these “marker” papers were used to develop full search strategies for each database. Keywords and index terms from these were adapted into full search strategies for each database and peer-reviewed via the PRESS checklist (37). All academic database searches were conducted between July and August 2024.

To capture unpublished work and grey literature, we conducted Google Scholar and advanced Google searches, and scanned relevant organizational websites, conference proceedings, policy documents, guidelines, best-practice documents, and toolkits. Reference lists of all records included were hand-searched to identify additional sources. We did not include “survivor research” or “mad studies” in our search terms since preliminary scoping indicated these traditions have limited representation in the forensic mental health literature (7). As theoretical orientations, they informed our conceptual framing and attention to power and epistemic injustice. The last search was conducted on January 31, 2025.

In addition to sources retrieved through these structured sources, we included findings from our broader integrated knowledge translation project, specifically a readiness assessment (26), relationship-building activities (34, 35), an ethnography (36), and an international community of practice. During the preliminary analysis, we also noted that many included documents provided limited guidance on health equity. As such, we selected two additional sources to further ground the emerging framework. Specifically, we integrated equity and engagement frameworks from the forensic field and broader health research, respectively (38, 39).

### 2.6 Screening process

All records retrieved from databases and grey literature searches were imported into Covidence (Veritas Health Innovation, Melbourne, AU), where duplicates were removed. After a pilot screening exercise, two independent reviewers screened titles and abstracts against predetermined inclusion criteria (see **Table 1**) and classified records as potentially relevant or excluded. Full texts of relevant citations were retrieved and assessed against the same inclusion criteria by the same pair of reviewers, with reasons for exclusion documented within Covidence. Any disagreements at either stage were resolved through discussion, with a third reviewer (the project PI) involved when needed; unresolved matters were brought to the broader project team for consensus. Consistent with rapid review practices, we did not calculate interrater reliability. Our emphasis was on timely synthesis and consensus/team-based resolution rather than statistical measurement of agreement. This review followed PRISMA reporting guidelines, and a PRISMA flow diagram summarizes the number of records identified, screened, excluded, and included (see **Appendix B**). In the end, 25 sources from the structured searches were included for data extraction, along with the six purposively identified sources described above, for a total of 31 records.

### 2.7 Data extraction

Data extraction followed a structured table that captured article characteristics and the following elements for each source: education and training components related to participatory research methods; compensation models; privacy and confidentiality approaches and policies; consent processes; plain language and other accessibility considerations; equity, diversity, and inclusion considerations; levels of engagement; approaches to evaluating engagement; and strategies for relationship and trust building. We also documented specific findings and outcomes associated with POR tools, along with any reported recommendations, challenges, barriers, or limitations encountered in their use. By “POR tools,” we mean any practical guidance, resources, and strategies described in the included sources, such as consent templates, engagement frameworks, remuneration frameworks, and patient advisory group structures. A list of all 31 included sources and associated extracted data are available in **Appendix C**.

### 2.8 Data analysis

**Figure 1:**
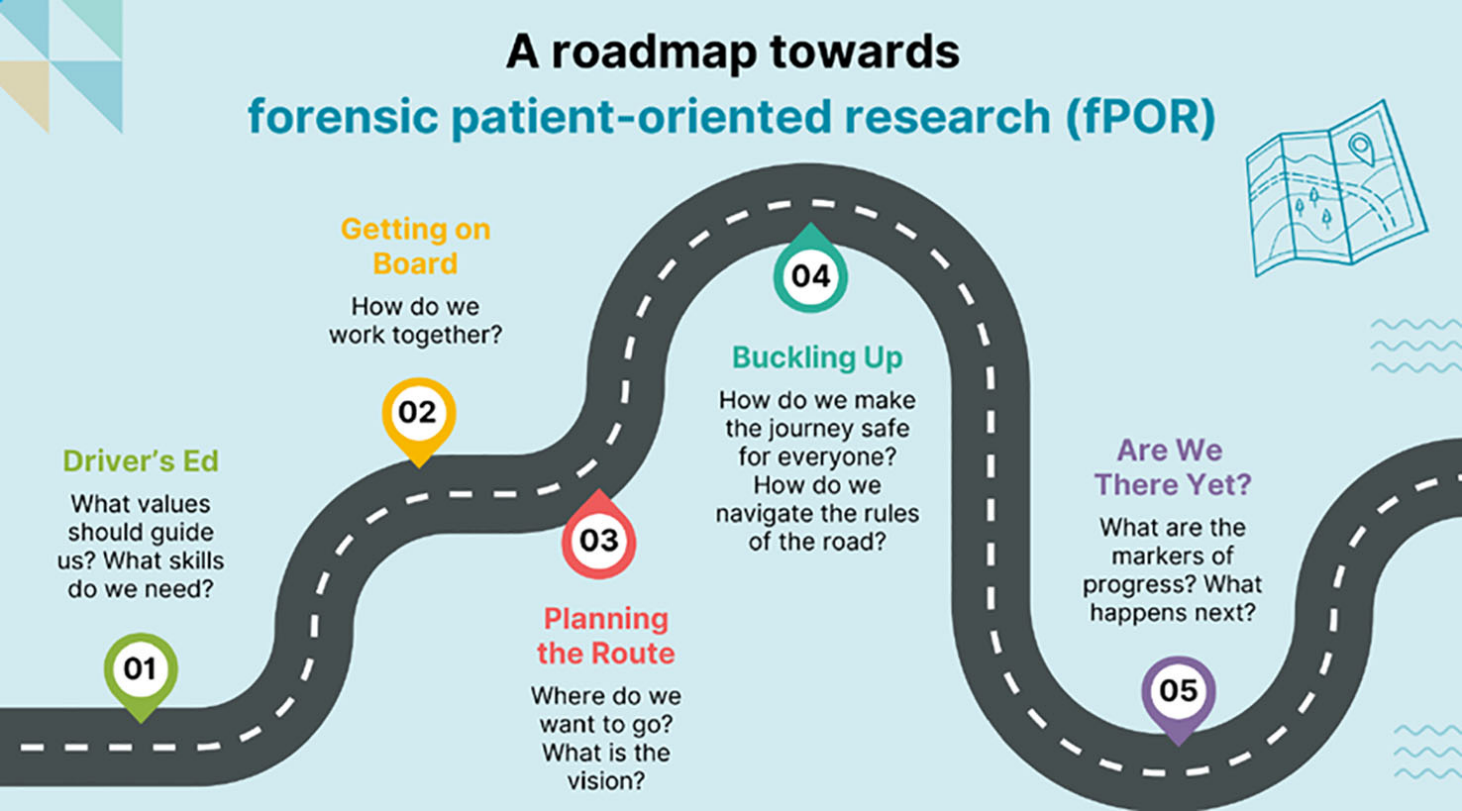
A roadmap towards forensic patient-oriented research.

Three team members (the Principal Investigator and two research analysts) conducted deductive content analysis (40) using an *a priori* framework derived from the knowledge exchange event. The initial framework was organized around five “roadmap” elements: 1) driver’s ed (values and skills); 2) getting on board (how to work together); 3) planning the route (vision and goals); 4) buckling up (safety, policies, and legal requirements); and 5) are we there yet? (markers of progress and next steps). See **Figure 1** of the initial roadmap framework developed in June of 2024.

Each reviewer mapped extracted data to the five categories described above, noting cross-cutting principles and values, such as trust, safety, and trauma-informed practice, that appeared across multiple elements. The three reviewers compared content analyses and syntheses and reconciled discrepancies through discussion until consensus was reached on how best to organize the data into the framework. During these discussions, the team debated whether the final guidance should follow a sequential, step-by-step structure or be organized by thematic dimensions, given the complexity and non-linear nature of conducting POR in forensic settings.

**Figure 2:**
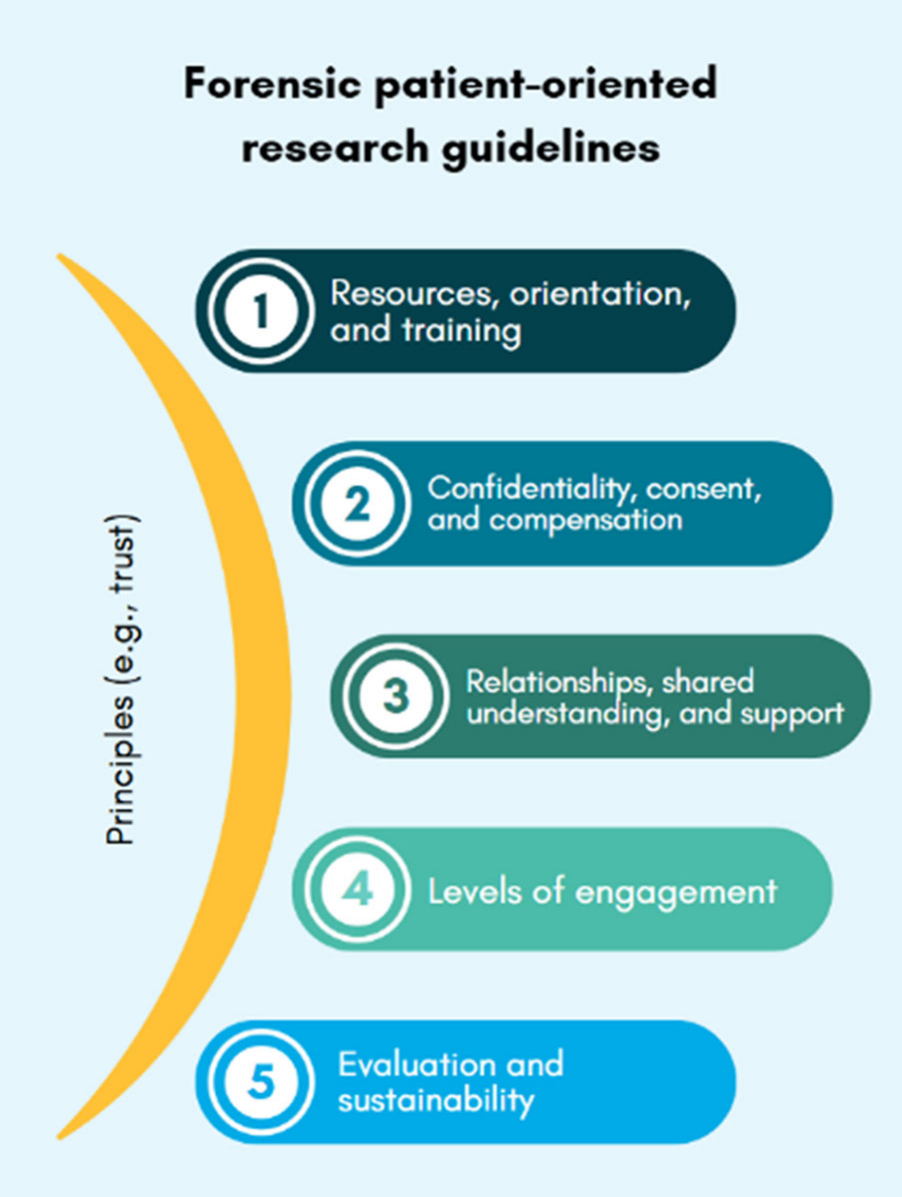
Forensic patient-oriented research guidelines: principles and dimensions.

Through an iterative process, the original roadmap framework was refined into a final five-dimension framework (see **Figure 2**), described in more detail in the findings below. Each reviewer was assigned one or two dimensions to synthesize, identify tools, resources, and guidance from the extracted data, and propose subheadings to organize content within each of the new dimensions. We did not use a formal quality appraisal or risk of bias assessment given the nature of rapid reviews of this kind, when the purpose was to map and integrate evidence to support guideline development rather than evaluate the effectiveness of interventions. We acknowledge that the included corpus contains a proportion of grey literature whose quality is variable, and that several sources originate from the authors’ institution. The inclusion of grey literature reflects our commitment to capturing practice-based knowledge, and the inclusion of our own sources reflects the project’s grounding in a local context. These biases were offset as much as possible by external validation through an international community of practice, project mentors from a different site, and patients.

### 2.9 Drafting the guidelines

The team integrated each of the syntheses into guideline sections titled “What and Why” for definitions and rationale, “How” for best practices and key considerations, and “Specific Tools and Resources” to support participatory practices. To enrich the syntheses, two reviewers integrated findings from project IKT activities into the themes from the literature, including qualitative interviews from the readiness assessment, lessons from the international community of practice, ongoing relationship building with patients and staff, and mentorship from University of Saskatchewan colleagues.

The draft guidelines were circulated to the project team, implementation team members, and patient advisors for feedback and validation, and then reviewed by forensic researchers and mentors, whose input helped ensure a clear, contextually grounded set of guidelines for conducting POR in forensic settings. Patient advisors conducted the final review and were asked to focus on clarity, relevance, and tone. Their feedback informed refinements to the structure, content, and accessibility of the guidelines.

Although this rapid review was complemented by earlier project activities as outlined above (e.g., readiness assessment, patient feedback), only published and publicly available sources are included in the synthesis of results below. Project findings and local learnings were used to support contextualizing these findings and supporting the refinement of the resulting guidelines.

## 3 Results

We identified 31 sources that informed the development of the five-dimension forensic POR framework (see **Figure 2**) and associated guidelines. Most sources were qualitative or mixed methods studies, case reports, frameworks, and policy documents focused on participatory or patient-oriented work in forensic and other secure mental health settings. Together with findings from earlier integrated knowledge translation activities carried out by the research team (26, 34–36) and equity and engagement frameworks (38, 39), these sources delineated core principles and strategies that were organized into five dimensions: 1) Resourcing, orientation, and training; 2) Confidentiality, consent, and compensation; 3) Relationships, shared understanding, and support; 4) Levels of engagement; and 5) Evaluation and sustainability. The five dimensions reflect areas of convergence between the academic and grey literature and the lived experiences and expertise of forensic mental health patients, staff, and researchers involved in this project. The literature provided a foundation for identifying core practices and principles, while patient advisors played a crucial role in interpreting how these practices could be operationalized in our setting. For example, as mentioned previously, patient feedback surfaced ethical tensions around sharing information with clinical staff and Review Boards, leading to a recommendation to co-create shared goals agreements that outline trusting ways of working together, approaches to maintaining privacy and confidentiality within the group, and ways of maintaining trust while being part of a research team that involves people occupying different positions of power. These issues were not always clearly reflected in the literature. The resulting synthesis therefore represents a co-produced interpretation of the evidence, grounded in both published literature and experiential knowledge. Below, we present the findings by dimensions, with cross-cutting principles as the opening section.

### 3.1 Principles

Across the literature and project activities, we identified several principles as being central to meaningful forensic POR: dignity, respect, transparency, trust, and accountability (6, 18, 26, 35, 41–43). Sources emphasize recognizing patients as individuals rather than defining them by their histories, and working in ways that explicitly counter stigma, structural discrimination, and epistemic injustice (7, 19, 24, 33, 42). Equity-oriented frameworks highlight the need to ask whose knowledge matters, who is and is not at the table, and to attend to racism, colonialism, criminalization, disability, gender, and poverty throughout project design, implementation, and evaluation (35, 38, 39).

Interdisciplinary teams and long-term, ongoing relationship building are necessary conditions for enacting these principles (6, 18, 22, 34, 35, 42, 44–46). Forensic POR is likely most effective when patients, hospital staff (including clinical, allied, and security staff), patient/client and family council members, and researchers collaborate as partners with clearly defined but flexible roles, supported by training, resourcing, and institutional backing, alongside open and ongoing communication about goals, processes, and limits and assurances that participation will not negatively affect patients’ legal status or care (18, 35, 42, 44, 47, 48).

### 3.2 Dimension 1: resourcing, orientation, and training

Forensic participatory research highlights the need for dedicated resourcing for patient engagement, staff and patient training, supervision, and research coordination, typically framed as allocating a defined portion of project budgets (e.g., around 10% or more) to cover compensation, training, coordination, resources, and relationship building activities (18, 41, 42, 48, 49). Studies and other reports emphasize that under-resourcing patient roles can lead to tokenism and inconsistent involvement, whereas sustained investment can enable equitable participation, flexible engagement, and diverse dissemination methods (e.g., plain language summaries, oral presentations, arts-based methods) (6, 17, 44, 48).

Time is also identified as a crucial resource. Existing work recommends allocating additional time for trust and relationship development, recruitment, preparation, navigating institutional processes, and adapting to unit routines, security incidents, and fluctuating patients’ mental health needs (7, 18, 22, 34, 45, 50, 51). Regular academic and funding cycles often conflict with the time needed to design and conduct meaningful participatory research, especially in secure environments (7, 18, 51).

Orientation and training findings indicate that all interest holders require role-specific preparation, with a strong emphasis on research training and capacity-building for patient partners (18, 26, 35). Recommendations include foundational research training and literacy (e.g., research cycle, methods, ethics, terminology), health and mental health concepts, forensic POR principles, and other related skills such as interviewing, data analysis, presenting at conferences, and digital skills (7, 22, 34, 44, 46, 47, 50). Participants in forensic settings often prefer experiential learning and modular training aligned with project milestones, offered in an ongoing way in multiple formats (group, one to one, and e-learning), adapted to literacy levels, access to technology, and learning styles, and reinforced through informal conversations, role playing, and iterative feedback (21, 22, 35, 42, 45, 46, 48). Training for staff and researchers should focus on trauma-informed practice, relational security, de-escalation, challenging stigma and discrimination regarding patients’ ability to be involved in research, and recognizing and addressing epistemic injustice (18, 21, 26, 35, 41).

### 3.3 Dimension 2: confidentiality, consent, and compensation

Confidentiality in forensic POR takes two forms: protecting patients as partners/advisors and as research participants, with clear, distinct descriptions of each role (35). Guidance documents stress the importance of co-developed confidentiality agreements, explicit boundaries around when confidentiality might be limited (e.g., imminent risk of harm), and clear assurances that contributions will not be entered into clinical records, shared with Review Boards unless requested, or used to influence levels of activity in the hospital (18, 26, 35, 44, 50). Sources caution that even anonymized data can be identifiable in small settings where patients often have long stays, and recommend that researchers offer pseudonyms, discuss authorship choices, and address fears about the dual roles that patients may hold as patients and research partners within a forensic mental health setting (18, 45, 47, 52, 53).

Consent in forensic POR has two aspects that should be clearly explained and maintained throughout the research process: consent to engage as a research partner or advisor and consent to participate as a research participant. Consent was consistently framed as a relational, ongoing process rather than a one-time activity (18, 45, 47, 48, 54). Evidence supports a presumption of capacity to consent, along with efforts to increase understanding through plain-language materials, multimodal information (written, verbal, visual), face-to-face conversations, and optional support from staff or peers, where preferred (18, 45, 48, 52–55). Regular check ins with patients are helpful to account for fluctuating capacity and circumstances, explicit separation of research and clinical roles, and safeguards to reduce perceived pressure to engage in research (e.g., neutral facilitators, statements that engagement will not affect care or legal status, and clear pathways for pausing or withdrawing from research engagement without negative consequences) (7, 18, 45, 47, 48). The literature also suggests that consent information be presented in plain language while avoiding jargon and preserving the original message (45). For example, researchers can use terms such as “agree” or “disagree” rather than “consent” (7, 18, 45).

Compensation guidance emphasizes fair, timely, and flexible recognition and suggests prioritizing monetary payment while attending to benefit implications (e.g., disability benefits), hospital policies and individual preferences, ensuring that pay rates, roles, and timelines are transparent and equitable across patient partners (7, 18, 35, 47, 50, 56, 57). The literature also suggests offering flexible forms of recognition. While monetary compensation should be provided to all patient partners and advisors and should be prioritized as a first option, some may decline or request alternative forms of recognition (35, 42, 56, 57). Alternatives may include opportunities for learning, co-authorship, peer connection, social activities, future employment potential, a sense of purpose, or a certificate of engagement (7, 42, 44).

### 3.4 Dimension 3: relationships, shared understanding, and support

The literature and our own project findings converge on the centrality of relationship-building as the foundation of forensic POR (18, 26, 34, 35, 42, 50). Sources describe the need for sustained researcher presence on units, informal encounters and conversations between researchers and patients, attendance at community meetings and hospital events, and tailored engagement (e.g., one-to-one versus groups) to build rapport and trust in environments characterized by high levels of distrust and structural power imbalances (22, 26, 34, 35, 47, 54). Working with staff allies while also clearly separating research spaces from clinical spaces was identified as key to negotiating security requirements and supporting engagement. Nevertheless, guidance cautions against relying on clinical staff as engagement leads because of risks of perceived coercion, potential gatekeeping, or conflicts of interest (18, 21, 42, 52, 58).

Shared understanding emerged as a distinct yet related theme, particularly with respect to clarity regarding roles, expectations, processes, and boundaries (35). Recommended strategies include early orientation and training sessions, co-developed terms of reference or “shared goals” agreements, consistent onboarding of new patient partners, use of meeting minutes to support continuity, and plain language, multimodal communication (e.g., visuals, glossaries, accessible formats) to reduce jargon and support diverse communication needs (7, 18, 35, 43–45, 50, 56, 57, 59).

Findings also highlight the need for proactive, individualized support plans that anticipate mental health, practical, and safety issues, including crisis and safety protocols, access to neutral support people, regular check-ins, debriefing after difficult discussions or situations, and attention to consultation fatigue (6, 18, 35, 44, 52). Peer mentoring and mutual support were identified in the literature as powerful mechanisms for sustaining engagement and mitigating the potential burden associated with dual roles as patients and co-researchers/research partners (22, 35, 45, 47, 58).

### 3.5 Dimension 4: levels of engagement

Patients in forensic settings will engage in research activities at varying levels over time, thus flexible engagement approaches are essential (6, 35). Frameworks detailing continuums of engagement (e.g., informing, consulting, involving, collaborating, empowering) were recommended to clarify expectations and even what is feasible in secure settings, align roles with preferences, learning stages, and capacities, and normalize shifts in engagement due to health, legal, or institutional constraints (6, 18). Involving patients as early as possible in setting research priorities and designing projects can lead to genuine power sharing, shared ownership, relevance, and trust. Some sources suggested including structured research priority setting exercises, open questions about current concerns, and dedicated projects to consult patients about ongoing research gaps and questions (6, 7, 42, 44, 46).

Current literature also highlights the importance of inclusive strategies for patients who cannot attend research meetings, such as sharing group insights one-to-one or using alternative formats to capture input (35, 54). Dissemination and implementation are also recognized as critical points for patient engagement. For example, research teams should involve patients in developing and delivering presentations, create accessible materials (e.g., lay and easy read summaries, art-based or other creative outputs), and present on project work at hospital or external events. Teams might also involve patients in implementation efforts and policy discussions to reinforce that research is intended to drive meaningful change (6, 35, 47, 51, 54).

### 3.6 Dimension 5: evaluation and sustainability

Evaluating forensic POR should extend beyond outcome measures to capture relational and experiential process dimensions, such as trust, psychological safety, power sharing, and perceived impact (7, 35, 44, 45, 52). Recommended practices include structured debriefing at different stages and at the end of projects, mixed-methods feedback (e.g., surveys, storytelling) for both patients and staff, and attention to accessibility and feelings of burden with engagement (35, 43, 47, 52). Evaluation is also framed in the literature as an important mechanism for project closure. It can be used to acknowledge endings, manage expectations, and facilitate reflection on the emotional and relational work that has occurred among patients and project team members (7, 26, 35, 42, 47).

To support sustainability, forensic POR requires institutional investment and cannot rely solely on individual projects or champions (6, 7, 18, 21, 51). Suggested strategies include maintaining expert patient and staff groups to support ongoing involvement and engagement, integrating knowledge translation and implementation support to embed findings into policies and practices, and creating pathways for continued involvement after discharge (e.g., alumni groups). We also heard that revisiting research priorities every few years with patient partners, and advocating for protected time, funding, and organizational structures, is important so that forensic POR is embedded as a standard way of doing research in forensic mental health settings, rather than an exception or afterthought (7, 35, 46, 47).

## 4 Discussion

The goal of this rapid review was to develop evidence-informed, context-specific guidelines for conducting POR in forensic mental health settings, addressing a gap in existing POR and forensic literature. We describe recommendations across five domains: 1) Resourcing, orientation, and training; 2) Confidentiality, consent, and compensation; 3) Relationships, shared understanding, and support; 4) Levels of engagement; and 5) Evaluation and sustainability. These recommendations are underpinned by cross-cutting principles of dignity, respect, equity, transparency, accountability, and relational approaches to trust building, learning, and research.

Many of the principles and recommendations discussed in this review are consistent with other guidance on participatory research in mental health. As noted above, existing guidance derives from a range of traditions, including survivor research (7–11, 44), patient and public involvement (15), community-based participatory research (60, 61), action research (62, 63), and POR (24, 64). Concepts such as power sharing, authentic engagement, and trust are frequently raised across these bodies of literature and align with the cross-cutting principles highlighted in the present review. Similarly, recommendations such as fair compensation, training, and peer support for patients are evident in broader guidance on participatory mental health research and in the forensic-specific literature reviewed here. As articulated by Faulkner, survivor research advances ethical and relational standards that have yet to be fully named or adopted in participatory forensic mental health research (7, 42, 44). Mad Studies offers a similar theoretical orientation that centers the epistemic authority of people who have lived experiences of mental illness and/or marginalization within mental health and related systems, although the forensic POR field has yet to fully draw on either tradition. We believe that bringing these approaches into conversation with one another is a necessary next step for the forensic POR field (65).

The case for meaningful patient engagement in forensic mental health research is also grounded in international normative frameworks. Canada has ratified the United Nations Convention on the Rights of Persons with Disabilities, which has direct implications for forensic mental health patients as members of research teams. Article 12 affirms the equal recognition of people with disabilities before the law and challenges assumptions that mental health-related disabilities justify diminished legal capacity or exclusion from decision-making, while Articles 19 and 29 affirm rights to community inclusion and public life (66). Anchoring forensic POR in this framework has the potential to position patient engagement as aligned with rights-based discourse. The five dimensions of our framework enact these commitments by emphasizing patient autonomy and agency, trust, and equitable forms of involvement at every stage of the research process.

However, foundational work on forensic POR has highlighted distinct considerations around security, layered stigma, coercion, risk management, and epistemic injustice. For instance, patients’ exercise of agency may be affected by fluctuating symptoms, involuntary and custodial treatment, and external or self-stigma (6, 18, 22). Fluctuating capacity is addressed in multiple dimensions of this review. For instance, dimension 1 notes that extra time should be built into projects to accommodate patients’ shifting wellness and capacity. Dimension 1 also calls attention to the historical roots of epistemic harm in forensic environments, that is, the ways patients have been minimized as knowers and how patients’ experiential knowledge is considered less valid in a field dominated by positivism and a narrow focus on individual risk. The forensic mental health field is rightfully starting to talk more about these epistemological considerations and their implication for participatory research practices (67). The second dimension also advises presuming capacity and checking in regularly to confirm a patient’s current ability to consent to participating as a team member or in research, while the fourth recommends creating flexible and accessible engagement options. Likewise, some of the distinct challenges of empowerment within an involuntary setting are addressed through recommendations to clarify the limits of confidentiality (dimension 2) and to consider and overcome potential distrust between patients and staff when working with institutional gatekeepers (dimension 3).

This review adds to the emerging literature on forensic POR by presenting a comprehensive, up-to-date, and practice-oriented synthesis. The first review on participatory forensic research was conducted in 2016 and provided an overview of the challenges and opportunities unique to forensic settings; it is widely cited as a foundational piece in the field (18). Previous literature has described challenges and approaches to forensic POR in the context of specific individual studies (22, 46) and/or elements of research participation, such as informed consent (48) or priority setting (6). A more recent scoping review brought together prison and forensic mental health studies and proposed next steps for reporting participatory research in journals to reduce tokenism (21). That review aligns with our findings on the need for closer attention to the ethical complexities inherent in forensic settings, gatekeeping among staff that can limit patient engagement and involvement, and resource constraints that contribute to limited uptake and sustainability of participatory methodologies (21).

A more recent systematic review published at the time of writing this paper synthesizes international evidence on participatory research involving forensic mental health patients and summarizes those findings into “active ingredients,” including, among many, funding, training, ethical considerations, informed consent, forming research groups, communicating in plain language, and building trust and rapport between patients and researchers (20). These ingredients of authentic forensic patient involvement validate core elements of our five-dimension framework, including the emphasis on plain language accessibility as a cross-cutting priority in dimension 2 (e.g., consent materials), adapted training formats in dimension 1, and multimodal communication tools and co-developed shared goals agreements in dimension 3. Ferra et al.’s “speaking the same language” theme extends dimension 3’s strategies for redressing power imbalances through relational communication, and the “impact for all” theme underscores the shared value and relational effects of participatory research, complementing Dimensions 4 and 5 of our guidelines, which translate these impacts into practical approaches to engagement, evaluation, and sustainability (20).

### 4.1 Limitations

One limitation of this review and our integrated knowledge translation approach is the narrow focus on forensic mental health settings specifically. We did not include literature from correctional or other settings that share characteristics with forensic contexts. This decision was motivated by our guideline development process specific to a mental health hospital, but it comes with potential epistemic costs. Correctional settings house large populations with significant mental health needs, and many principles in our framework likely have relevance there. Future projects should examine the transferability of these guidelines across custodial contexts building on other synthesis work that has bridged forensic and correctional settings (21). We also acknowledge that the specific nature of forensic care varies across jurisdictions. While knowledge users engaged throughout this project included researchers in other provinces and countries, patients and clinicians who provided input (including authors of this paper) were based in a single setting in Ontario, Canada. This inevitably influenced our interpretation of the findings and the structure of the guidelines.

### 4.2 Future directions

Future directions include implementing, evaluating, and adapting the guidelines informed by this review, and reporting on the extent to which patients actively contribute to participatory research practices (18, 19). While a growing number of forensic research teams internationally are adopting participatory practices, forensic POR remains an emerging field. Moreover, formal evaluations of participatory practices in forensic settings are scarce. Opportunities exist to develop theory-driven evaluations that articulate the objectives and observed effects of forensic POR. This may include using tools that measure patient engagement, such as the Secure Quality Improvement Patient Tool (59) or the Public Engagement Evaluation Tool (68), as well as analyzing barriers and facilitators to implementing the practices described in this review. The recommendations of this review may also require adaptation to address contextual heterogeneity arising from variations in security levels or in the institutional and legal contexts that structure forensic settings.

Finally, it is critically important that forensic patients lead ongoing work to advance forensic POR. Our research team includes a current patient and additional patients who provided consultative input into the guidelines. It is our intention that the review and accompanying guidelines support the ongoing shift towards greater breadth and sustained patient engagement in forensic POR in our own setting and beyond. Our goal of this work remains to increase visibility of forensic patient expertise and create more opportunities for patient leadership across the field of forensic POR as it matures.

## 5 Conclusion

Our rapid review of the literature and integrated knowledge translation approach builds on current research by translating evidence into guidelines tailored for forensic hospitals and similar secure settings. The guidelines frame forensic POR as a practice that can support ongoing global efforts to more meaningfully engage forensic patients in research. As participatory research practices continue to be adopted, taking stock of new and emerging knowledge is key to informing practice and ensuring that guidance remains anchored to the specificities of forensic settings and the needs of knowledge users, including patients, health care providers, and researchers working within those settings.

## Data Availability

The original contributions presented in the study are included in the article/**Supplementary Material**. Further inquiries can be directed to the corresponding author.
