# Supplementary figures and images for: Developing forensic patient-oriented research guidelines: a rapid review using an integrated knowledge translation approach
Source: Front Psychiatry. 2026 Jul 2;17:1805912. doi: 10.3389/fpsyt.2026.1805912 (PMC13373535; doi:10.3389/fpsyt.2026.1805912)

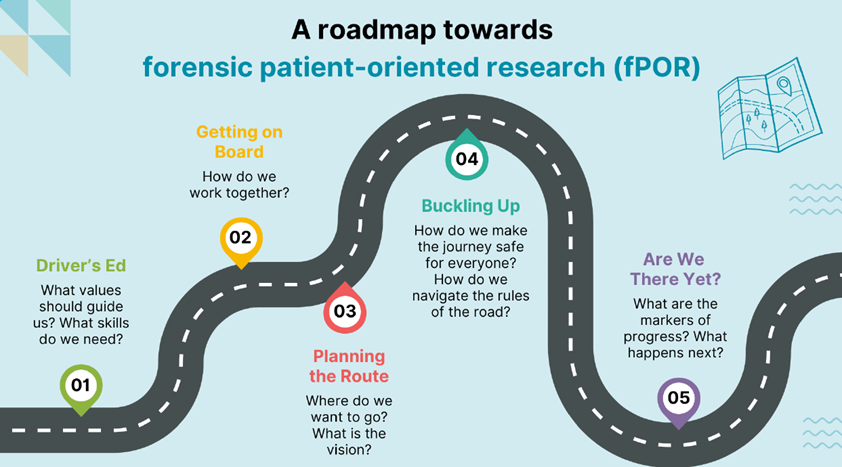

Supplement: Supplementary file 1 [file Image1.png]

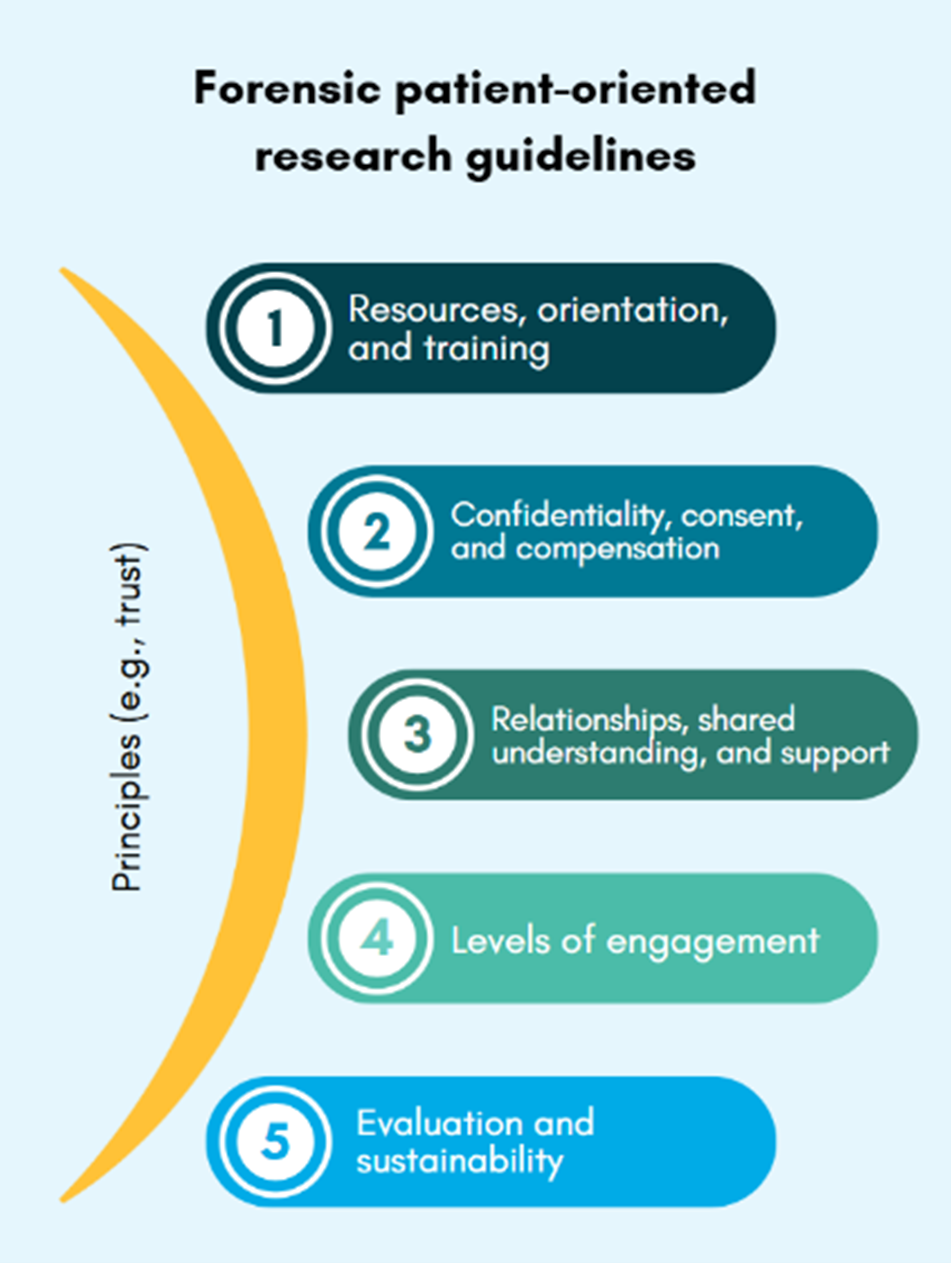

Supplement: Supplementary file 2 [file Image2.png]
