# Supplementary material for: Developing forensic patient-oriented research guidelines: a rapid review using an integrated knowledge translation approach
Source: Front Psychiatry. 2026 Jul 2;17:1805912. doi: 10.3389/fpsyt.2026.1805912 (PMC13373535; doi:10.3389/fpsyt.2026.1805912)

Supplementary Material

**Appendix A: Sample search strategy using Medline (OVID)**

|  | **Searches** | **Results** |  |
| --- | --- | --- | --- |
| 13 | 7 and 12 | **576** |  |
| 12 | 8 or 9 or 10 or 11 | 59828 |  |
| 11 | exp Patient Participation/ or exp Community-Based Participatory Research/ | 35305 |  |
| 10 | "patient and public involvement".mp. | 1527 |  |
| 9 | (community-based participatory research or participatory action research or action research or peer researcher* or patient researcher* or patient partner* or consumer researcher* or co-design* or codesign* or co-production or coproduction or co-create* or cocreate*).mp. | 21290 |  |
| 8 | ((patient or client or user or consumer or family carer or caregiver) adj2 (oriented or focused or centered or led or engag* or involv* or participat* or collaborative or inclusive or "lived experience") adj5 (research or trial or study or method* or process or design)).mp. | 10997 |  |
| 7 | 1 or 2 or 3 or 4 or 5 or 6 | 56322 |  |
| 6 | "insanity defense".mp. | 1908 |  |
| 5 | "criminal responsibility".mp. | 692 |  |
| 4 | "not guilty by reason of insanity".mp. | 202 |  |
| 3 | "not criminally responsible".mp. | 101 |  |
| 2 | ((secure or forensic* or offender*) and (psychiatr* or hospital or ward or institution or nursing or mental disorder* or mental* ill* or mental health)).mp. | 25661 |  |
| 1 | exp forensic psychiatry/ or exp "commitment of mentally ill"/ or exp Insanity Defense/ | 40706 |  |

**Appendix B: PRISMA diagram**


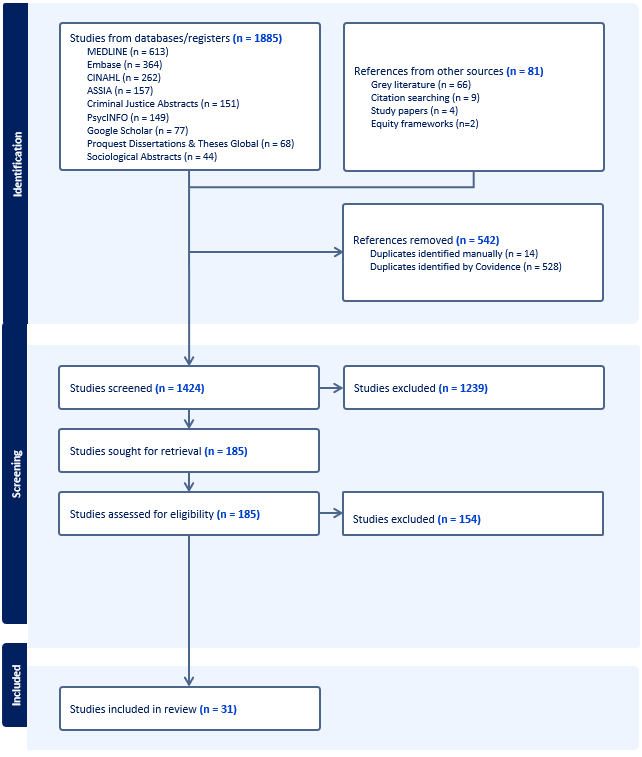

Supplement: Supplementary file 3 [file Table1.docx]
